# Active Prospective Control Is Required for Effective Sensorimotor Learning

**DOI:** 10.1371/journal.pone.0077609

**Published:** 2013-10-23

**Authors:** Winona Snapp-Childs, Elizabeth Casserly, Mark Mon-Williams, Geoffrey P. Bingham

**Affiliations:** 1 Department of Psychological & Brain Sciences, Indiana University, Bloomington, Indiana, United States of America; 2 Institute of Psychological Sciences, University of Leeds, Leeds, United Kingdom; VU University Amsterdam, The Netherlands

## Abstract

Passive modeling of movements is often used in movement therapy to overcome disabilities caused by stroke or other disorders (e.g. Developmental Coordination Disorder or Cerebral Palsy). Either a therapist or, recently, a specially designed robot moves or guides the limb passively through the movement to be trained. In contrast, action theory has long suggested that effective skill acquisition requires movements to be actively generated. Is this true? In view of the former, we explicitly tested the latter. Previously, a method was developed that allows children with Developmental Coordination Disorder to produce effective movements actively, so as to improve manual performance to match that of typically developing children. In the current study, we tested practice using such active movements as compared to practice using passive movement. The passive movement employed, namely haptic tracking, provided a strong test of the comparison, one that showed that the mere inaction of the muscles is not the problem. Instead, lack of prospective control was. The result was no effective learning with passive movement while active practice with prospective control yielded significant improvements in performance.

## Introduction

Action theory was formulated in the 1980's, inspired by results that revealed intrinsic relations between kinesthesis (or somatosensation) and motor control (for reviews, see [1], [2]). On the one hand, psychophysical results showed that kinesthesis is significantly better in the context of actively controlled posture and movement [3], [4], [5], [6]. On the other hand, investigations of motor control showed that somatosensation was intrinsic to the control of joint posture and movement [7]. This was captured in the Equilbrium Point (or Lambda) model (for reviews, see [8], [9], [10]). These results showed that perception and action are co-dependent. An implication was that movements are not passive products of causal stimulation (reflexes) or causal commands (motor programs), but are instead, actively generated and emergent through the interactions of both efferent and afferent elements. Accordingly, effective motor learning would entail actively generated and controlled movement.

In the 1990's, these observations on the essentially sensorimotor nature of movement also led to the development of model based theories of the control of actions (e.g. [11], [12]). Using insights from the ‘Smith predictor’ in control theory, Miall and collaborators [13] hypothesized that inverse models of movement system dynamics are used to predict actual sensory feedback to enable significantly more stable sensorimotor control. Sensorimotor learning would require the development of such inverse models and this, in turn, would require active generation and control of movement to expose the system dynamics to be captured by the inverse model. These alternative theories (that is, Equilibrium Point and Inverse models) remain prominent in the movement literature (e.g. [14], [15], [16]). Both predict that active control of movements should be required for effective sensorimotor learning.

This expectation is also consistent with Newell's [17] observation that learning a novel motor skill requires production of qualitatively appropriate movement that can be fine-tuned (see also [18]). An actively generated approximation to the desired movement is successively improved through subsequent sensorimotor practice. A need for active generation and control of movements presents a major hurdle for segments of the population with movement disabilities, for instance, Developmental Coordination Disorder, Cerebral Palsy or stroke [19], [20]. These people are typically unable to achieve the requisite initial approximation of a desired movement and are unable to improve through practice of actively generated movements. A traditional approach used by movement therapists to overcome this problem is to model desired movement skills for the learner with the hope that the learner might begin to approximate some form of the required skill and then, proceed to improve it through practice. Accordingly, a therapist will move the limbs of the learner passively through a desired form of movement (called “active assist”). Similarly, robotic approaches to therapy have been developed that replace the therapist with a robot that moves the passive limbs of the learner through the movements to be acquired (for a review, see [21], [22]). Generally, such robotic approaches to therapy have not been found to be effective [22], [23], [24]. Wong, Kistemaker, Chin, and Gribble [25] did find that passive motion of the arm and hand along a circular, constant speed target trajectory improved learning of the movement, but the learning failed to generalize beyond the exact trajectory that was passively experienced. When they tested movement around the circle in a direction opposite to that experienced during training, no learning was exhibited. Beets, Macé, Meesen, Cuypers, Levin, and Swinnen [26] investigated passive training of a novel bimanual rhythmic coordination, namely, simultaneous oscillations of left and right wrists in a 90° phase relation with a 2∶1 frequency relation. They found no learning of the 90° relative phase as a result of the passive training. Participants did exhibit an ability to produce the 2∶1 frequency relation but, as the authors acknowledged, special training is not really required to achieve this.

Why is passive training ineffective? One possible reason is because the muscles are inactive, rendering the sensory support for control of the muscles (e.g. muscle spindles and golgi receptors in tendon) also inactive and thus, ineffective. This is consistent with the insights that inspired the development of action theory as well as with results from studies on proprioception [3], [4], [5], [7], [8], [9], [10], [14], [15], [27], [28], [29], [30]. In the performance of actions, the current state of the motor apparatus must be perceived relative to the constraints imposed on the action by the environment to allow effective control of movement to complete a task. Effective proprioception is essential for this. An example is portrayed in Cole's *Pride and a Daily Marathon* [31], which provided an account of a young man who lost all large fiber afferents below the neck and thus, kinesthesis. With this, he lost the ability to control his movements. In time, he regained motor abilities by substituting use of vision for lost kinesthesis to perceive the ongoing evolution of his own movements relative to his surroundings.

However, especially in the case of visually guided actions, the problem involves more than quiescent musculature. To allow appropriate adjustments in motor control, visually guided actions entail detection of visual information about and perception of environmental circumstances that will be encountered in the near future, that is, visual anticipation (e.g. [32], [33], [34], [35], [36], [37], [38]) or prospective control (e.g. [39], [40], [41], [42], [43], [44], [45]). Control of interceptive actions (e.g. [39], [40]) and steering of locomotion (e.g. [46], [47], [48], [49], [50]) are extensively studied paradigm examples. To catch a ball, the actor has to perceive, first, where the ball is traveling so as to be at the appropriate location to intercept it and then, second, when the ball will arrive at that location, so that the catching action can be initiated sufficiently in advance to successfully catch the ball. Alternatively, to steer, an actor has to perceive the path ahead to initiate control of turning sufficiently in advance of a curve, for instance. Similar prospective control is required in actions like cursive handwriting on lined paper where the approach of the pen to a line must be visually anticipated to initiate control of the curved loop at the top of a letter. In fact, most any action requires prospective control entailing perception of the current state of the limbs and motor system in relation to the surroundings (including the rest of the body) to be able to control movements appropriately. Perhaps it is the absence of prospective control, then, that renders passive movements ineffective for sensori-motor learning. To test this possibility requires a form of passive movement in which prospective control is absent, but the muscles are not quiescent. These requirements are met by haptic tracking movements.

Therapists sometimes use a form of haptic tracking to help move a learner through a desired movement. The learner grasps the therapist's finger, for instance, and then moves to keep the felt pressure of the finger in the grasp constant as the therapist moves his or her finger through a trajectory. The result is that the learner moves his or her arm and hand along a desired movement path. In this case, the muscles are active, but the form of the movement is determined by the therapist and the guidance of such movements is not based on future-specific information, that is, prospective control. So, the movement is passive in the sense that the learner is not prospectively controlling the movements to be learned despite the muscles being active. We investigated whether this type of passive practice would yield effective sensori-motor learning. If passive practice is ineffective due to quiescent muscles (and poor sensory information as a result), then use of haptic tracking tasks might provide a good therapeutic alternative to the modeling of movements in a strictly passive way. On the other hand, if passive practice is ineffective for reasons beyond peripheral sensorimotor inactivity, then this would be an important fact to reveal. If haptic tracking is found to yield ineffective sensorimotor learning, then it becomes clear that prospective control is an essential component of effective motor learning and furthermore, that this is the meaning of active movement and its importance for motor learning.

The effectiveness of training using haptic tracking has been investigated in two related studies of motor learning [25], [51]. Both studies tested learning to move the hand along a circular path. Both studies reported that practice using haptic tracking was relatively ineffective. Lui, Cramer, and Reinkensmeyer [51] reported that it was equally effective as mere visual specification of the target path shape. Therapists also model movements by simply demonstrating them to provide visual specification of the movement form. However, this approach provides the weakest form of assistance for learners who have difficulties even getting started in producing given movements (e.g. CP, DCD, stroke). On the other hand, moving the hand along a circular path is pretty easy to do depending on the required accuracy with which a circular path should be produced. Haptic tracking might indeed be a poor way to train to produce a perfectly circular movement path (as compared to one that is a close approximation). Nevertheless, haptic tracking might be a good technique for learning types of movements, like moving along a circle versus a spiral versus a series of loops. On the other hand, it might not if good prospective control is required.

If haptic tracking fails, then what alternative might there be for the movement therapist? The emerging realization is that improved motor learning requires active movement generation with support that puts the learning into the ballpark [52], [53]. One way to do this is to use forcing that is proportional to movement errors relative to the goal of movement. The forcing puts the movement back onto a target path. Scaling of the forcing relative to the errors yields a gradual reduction of the support for accurate movements as movement control improves. Marchal-Crespo and collaborators [52] investigated this approach in the context of a steering task. Participants manipulated a steering wheel to steer a virtual vehicle around a course along a curved roadway. The result was good learning consisting of improved prospective control. Analyses revealed that participants learned to initiate turning earlier in anticipation of perceived curves in the roadway, that is, they acquired good prospective control. A similar result has been obtained by Milot et al. [54] in the context of a discrete button pushing task.

So, the key to improved sensori-motor learning may be to provide support to the active generation of voluntary movements that keeps the movements within a good approximation of the targeted skill and then, gradually to reduce the support as movement improves. This was the approach used by Snapp-Childs et al. [53] who developed a method for training manual actions performed by children with Developmental Coordination Disorder. The method required the children to generate limb movement actively to move their hand along a target 3D path, while providing support that allowed the children to perform the movements as well as typically developing children. The task entailed prospective control with support. The method was designed to allow the children with Developmental Coordination Disorder to produce qualitatively appropriate movements so as to be able to refine their movements subsequently. As the children practiced, the level of support was gradually reduced while allowing the children to maintain their level of performance. In the end, the children with Developmental Coordination Disorder exhibited substantial improvement to perform as well as the typically developing children who had also practiced at the task.

The task was one that would normally be extremely difficult, if not entirely impossible, for children with Developmental Coordination Disorder. A stylus was used to move a bead along a 3D wire path that looped around to bring the bead back to where it began. The difficulty was in keeping the tip of the stylus in contact with the wire while pushing the bead. All participants find this task to be difficult including typically developing children and adults [53]. Support was provided by making the wire magnetically attractive to the stylus, so as to hold the stylus onto the path. The level of support was manipulated by varying the strength of the magnetic attraction. At the end of training, all participants were able to perform the task well with no support.

**Figure 1:**
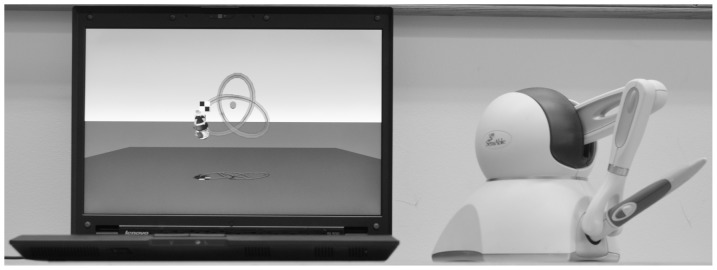
The PHANTOM Omni with the display.

In the current study, we compared these two methods of training, namely, haptic tracking and active movement with support. We investigated whether haptic tracking as a form of passive practice would yield effective motor learning. We used a haptic virtual reality device, the PHANTOM Omni (Sensable Technologies Inc.), which interfaced with a computer-generated display, to train healthy young adults to perform a novel sensorimotor task. See Figures 1a and 1b. One group received active training, where the stylus could be prospectively controlled by the participant, and another group received training with haptic tracking, where the stylus was moved automatically by the PHANTOM. We then compared the performance of these two groups with each other and to a control group that did not receive training.

The goal of training was not to have participants learn to produce any particular movement *per se*. Rather, the goal was to train participants to produce movement trajectories with proper amounts of compliance and to anticipate changes in curvature and torsion along a proscribed path; that is, to prospectively control their movements. We expected that the active training group would improve due to training while both the passive haptic tracking group and the control group would fail to exhibit learning. We also predicted that the active training group would exhibit generalization of training to novel movement paths, but that the other two groups would not. The reason is that we expected the active group to acquire good prospective control of such movements in general whereas the other groups would not. In particular, training using haptic tracking does not entail prospective control and thus, should not yield good prospective control in a skilled task that requires it. (For an initial report of this research see Bingham et al. [55].)

## Methods

### Participants

Thirty-six adults participated in this experiment. Twelve participants were assigned to one of three groups (active: 5 females, 7 males, 20–35 years old; passive: 7 females, 5 males, 22–35 years old; control: 8 females, 4 males, 20–32 years old). All of the participants reported normal or corrected-to-normal vision, no history of motor or neurological impairments, and (except for two) right hand preference. All participants used their preferred hand in the testing and training phases of the study.

### Ethics Statement

This study was approved by the Institutional Review Board at IU Bloomington and all participants gave written informed consent.

### Apparatus

Participants interacted with a 3D display by moving a handheld stylus. The display was presented on a 15” computer screen that was located on a desk 70 cm from participants. The stylus was attached to a desktop force feedback haptic virtual reality device, a PHANTOM Omni (Sensable Technologies, Inc.) and was located 50 cm in front and 10 cm to the right (or left for left-handed participants) of the computer screen. No support was provided for the hand/arm with which participants were performing the required tasks (i.e. the dominant hand/arm).

The PHANTOM is an impedance control device [28] where the user moves the stylus and the device reacts with a force if a virtual object is encountered (the PHANTOM thus has displacement as an input and force as an output). The mass and friction of the actual PHANTOM has been made small by careful mechanical design using cables driven by high performance DC motors. In the baseline trials and the practice trials experienced by the active group, a force was programmed so that the stylus was attracted to the path if it moved away from the 3D spatial locations that specified the path (at a phenomenological level as if a ‘magnetic’ force were present). The force pulling the stylus was modeled as a virtual spring where the stiffness of the spring could be altered. The spring had a virtual length of ≈0.5 cm from the center of the path so the force dropped to zero if the (virtual) stylus moved >0.5 cm from the (virtual) path. The spring stiffness was parametrically varied to alter task difficulty. The forces pulling the stylus towards the spring were set at six different levels corresponding to forces of approximately 2.02N, 1.08N, 0.83N, 0.57N, 0.35N and 0.13N.

The PHANTOM was also programmed to provide training trials to the passive group where the group did not need to actively control the stylus. In these trials, the endpoint of the stylus was moved around the 3D path by the PHANTOM (i.e. we inverted the normal impedance control and used the PHANTOM as an admittance controlled device). This was achieved by programming the endpoint (visible to the participant as a red bead) of a virtual spring to move around the path at a prescribed speed. The spring was set with maximum stiffness (approximately 3.3N) and this generated enough force to move the stylus without human intervention. If the stylus is held in this programming configuration, it feels as if there is a guiding force helping the hand around the path (it was described by some participants as the ‘mother's hand effect’ as it is reminiscent of a parent guiding a child's hand when first learning to write). It was obviously possible for participants to exert enough force to stop the stylus moving but simply relaxing the arm musculature and supporting the weight of the stylus provided the experience of moving efficiently and accurately around the path (as shown by the movement of the red bead around the path).

### Procedure

All participants performed the same basic 3D tracing tasks during two sessions (baseline and post-training) separated by one to two weeks. The separation varied slightly across participants as it depended upon the availability of the participants (when it was convenient for them to attend for testing) but there were no group differences in the average time between sessions. Once centered with respect to the computer screen, participants were instructed to grasp the PHANTOM stylus as if they were grasping a pencil. The task was to use the PHANTOM to move a virtual stylus and control its endpoint to push a virtual red bead along a virtual 3D path visible in a computer graphics display (see Figure 1a and 1b) from a starting location (the solid square) to a finish (the checkered square). The participants were asked to push the bead, from the starting location to the finish point, as quickly as possible. If participants deviated from the path, they had to return the stylus to the path at the location along the path where they had left it to continue pushing the bead to the finish point. This was explained to them. They were given no other explicit goals regarding accuracy. Trials ended either when the bead reached the finish point or 90 s had elapsed from the start of the trial. The task was modeled on a wooden maze toy. The toy is commonly found in pediatrician waiting rooms consisting of color beads on roller coaster like colored wires attached to a wooden base. The goal is to move a brightly colored bead from one end, through a series of twists and turns, to the other end. However, instead of being able to use one's fingers directly, a pencil must be used to move the bead along the wire path.

**Figure 2:**
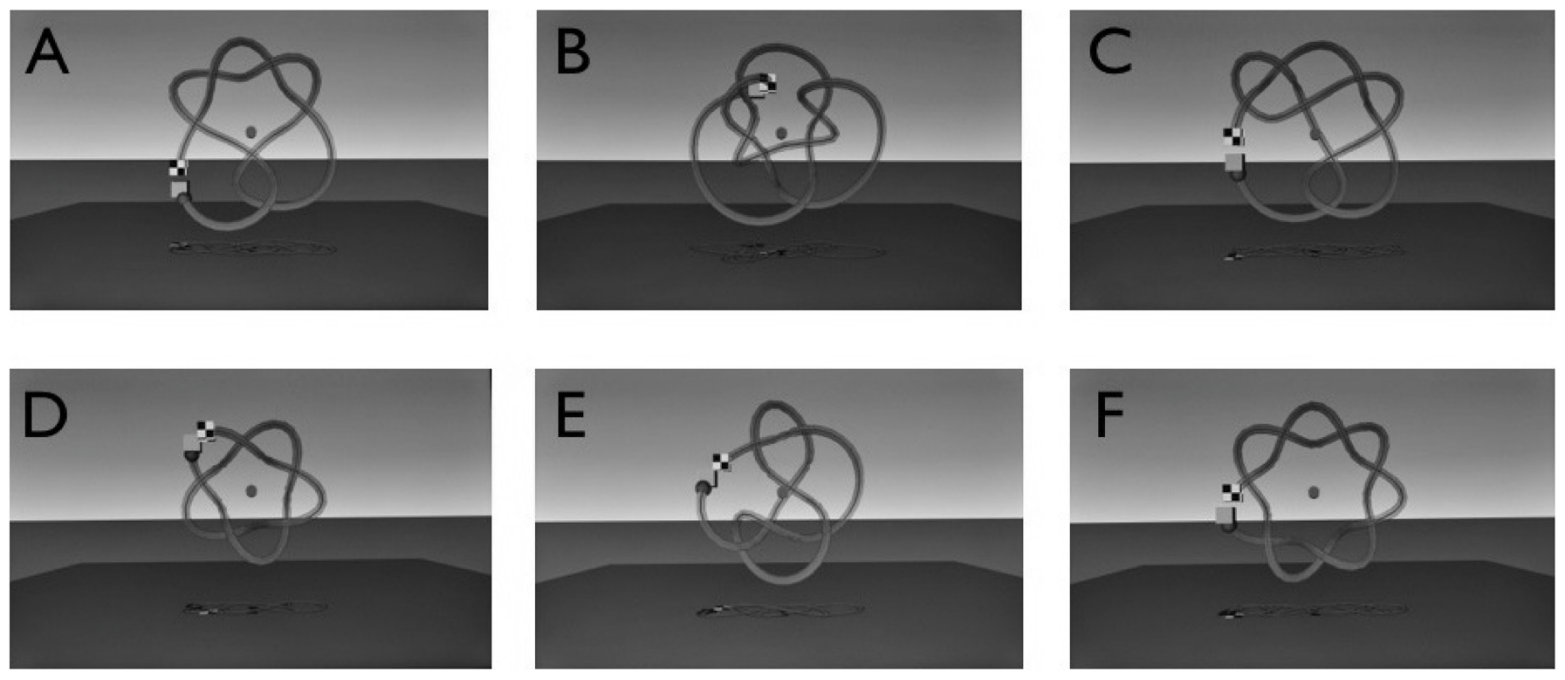
Paths used during the experiment. A) Baseline Path, B) Transfer Path 1, C) Transfer Path 2, D) Training Path 1, E) Training Path 2, F) Training Path 3.

During the first session, participants performed two trials at each of six levels of support (12 total trials) on the Baseline Path (113 cm in length, pictured in Figure 2a). The order of trials was fixed so that participants started with the highest level of support and progressed to the lowest. In order, spring stiffness values associated with the highest to lowest levels of support were: 2.02N, 1.08N, 0.83N, 0.57N, 0.35N and 0.13N. These stiffness values (and associated levels of support) correspond to variations in task difficulty and have been used in previous research (see [53]). Before one has acquired skill in performing this task, it is an extremely difficult task that elicits much frustration when attempted without any support, that is, with low stiffness. Participants show little improvement even after extended practice. (This is especially true of individuals with Developmental Coordination Disorder). On the other hand, the goal of the task is to be able to move the bead from one end of the wire to the other end rapidly without any support. During the final session, participants performed the same set of trials as during the first session plus an additional two trials on each of two novel paths at the lowest level of support (16 total trials). The novel paths are referred to as Transfer Paths (Transfer Path 1: 131 cm in length; Transfer Path 2: 124 cm in length) and are depicted in Figures 2b and 2c, respectively. These paths were chosen because they were longer paths with more extensive changes in curvature and torsion when compared to the Baseline path – that is, they were more challenging. Performance on these paths allowed us to examine generalization of learning to more difficult conditions.

In between these sessions, participants in the active and passive groups received training on a different set of paths (see Figures 2d, 2e, 2f; in order, paths were 84 cm, 107 cm, and 116 cm in length). Participants in the control group did not receive any training. There were three training sessions for the active and passive groups. In each training session, participants in the active group performed two trials per path for each of six support levels (the same levels as in the baseline and post-training assessments) in a fixed order (36 training trials). The participants in the active group were asked to push the bead, from the starting location to the finish point, as quickly as possible. Participants in the passive group completed the same number of trials as the active group participants. However, the participants in the passive group were moved by the PHANTOM, from the starting location to the finish point, at a fixed duration of 15 s. That is, once the stylus reached the starting location, the virtual stylus and red ball moved automatically along the path while the participant held the PHANTOM stylus. Thus, participants in the passive group did not experience any variations in stiffness and did not have to generate their own movements whereas the active group did.

### Data analysis

The three dimensional Cartesian coordinates of the virtual stylus tip (corresponding to the red bead visible to the participants) were recorded at 50 Hz. These data were filtered using a dual pass second order Butterworth filter with a 5 Hz cut-off frequency. Using these data with the known coordinates of the target trajectory (the path) we computed both *temporal* and *spatial* measures of performance. Trial duration was computed as a temporal measure. Trial duration was the time it took for a trial to be completed (the time in seconds from when participants arrived at the starting location to when they arrived at the finish marker). We selected duration because it provides a single unambiguous global measure of performance that related directly to the explicitly stated goal of the task. Moreover, duration is typically used as a performance measure in a wide range of motor tasks [56]. We also examined two *spatial* kinematic performance measures both of which reflected positional error: frequency off path and path length. Especially with low spring stiffness, participants tended to come off the path and this cost time (the time required to re-position the stylus). Frequency off path was simply the number of times per trial that this happened (from when participants arrived at the starting location to when they arrived at the finish marker). Path length was the total distance traveled (in cm) in a trial by the participant controlled stylus (from when participants arrived at the starting location to when they arrived at the finish marker).

We averaged the dependent measures separately for each participant, over the trials performed in a given condition (level of spring stiffness, path) and session (baseline versus posttest). We also computed learning scores by subtracting post-training performance from baseline performance. Statistical analyses of the group differences and changes in the dependent measures were performed with mixed design and single factor analysis of variance. For these analyses, group (active, passive, control) was a between-subjects factor; support level (that is, level of spring stiffness which varied from 0.13N to 2.2N), session (baseline vs. post-training), and path (Baseline path vs. Transfer paths) were within-subjects factors. Regression analyses were also performed to examine the relationship between the temporal and spatial measures.

## Results

### Temporal measure

We recorded the durations for completion of movements tracing these 3D wire paths. We first confirmed that there were no differences among the groups tested before training. We expected that active training should yield improved (shorter) durations in post training trials. This expectation was confirmed by the results. We expected no improvement by participants that trained using passive haptic tracking or by participants who did not train (controls). This expectation was not confirmed strictly. The passive and control groups did exhibit improvement in a comparison of baseline with post training trials. However, these latter groups exhibited exactly the same improvement. Therefore, because the participants in the control groups did not train, we concluded that the passive training failed to yield sensori-motor learning. Instead, baseline trials that were of necessity performed actively yielded the improvement exhibited by both the passive training and control groups. Also, two passive groups trained using different imposed durations. This yielded no difference in post training performance. Finally, we tested transfer trials and found evidence of learning only for the active training group.

**Figure 3:**
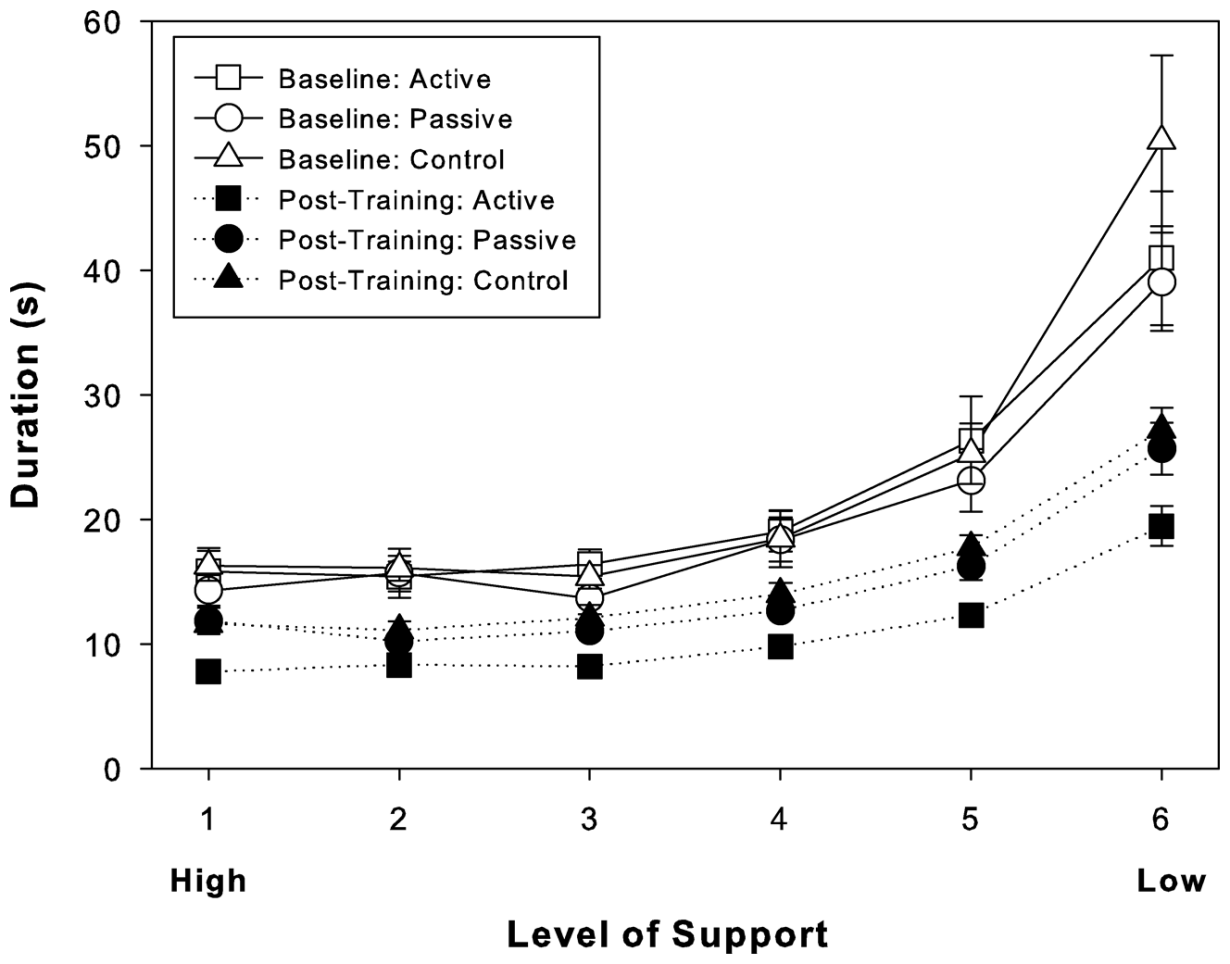
Mean trial durations for the Baseline Path by support level (1 = high = high spring stiffness; 6 = low = low spring stiffness), group, and session.

First, we examined levels of performance across the three groups before training (see Figure 3). As expected and as revealed by the mixed design ANOVA, there were no differences between the groups at baseline (group: p>0.5, group by level of support interaction: p>0.4). Also as expected, mean trial durations increased as the level of support decreased (support level: F(5,165) = 63.6, p<0.001).

Next, we examined differences in learning scores (baseline – post training). As shown in Figure 3 and contrary to expectations, there were improvements in performance for all groups, although the amount of improvement varied by group as well as by support level. As revealed by mixed design ANOVA, the active group exhibited more improvement than did either the passive or control groups (group means were as follows: active  = 10.5 s; passive  = 6.1 s; and control  = 6.7 s; main effect of group: F(2,33) = 3.6, p<0.04). The greatest improvement occurred at low support levels (support level: F(5,165) = 15.2, p<0.001), but this was consistent across groups (no group by support level interaction: p>0.9). Next, we tested the groups taken two at a time. In all cases, support level was significant (F(5,110)≥ 9.1, p<0.001) and the interaction was not (p>0.5). Active was different from both passive (F(1,22) = 6.2, p<0.02) and control (F(1,22) = 4.7, p<0.04), but passive and control were not different from one another (p>0.7). When these analyses were performed on the post-training durations shown in Figure 3, the ANOVA on three groups yielded significant main effects for group (F(2,33) = 17.4, p<0.001) and support level (F(5,165) = 110.7, p<0.001), but no interaction (p>0.4). In analyses on groups comparing them two at a time, support level was significant (F(5,110)>68.0, p<0.001) and the interaction was not (p>0.1) in all cases. Active was different from both passive (F(1,22) = 22.8, p<0.001) and control (F(1,22) = 32.6, p<0.001), but passive and control were not different from one another (p>0.2).

**Figure 4:**
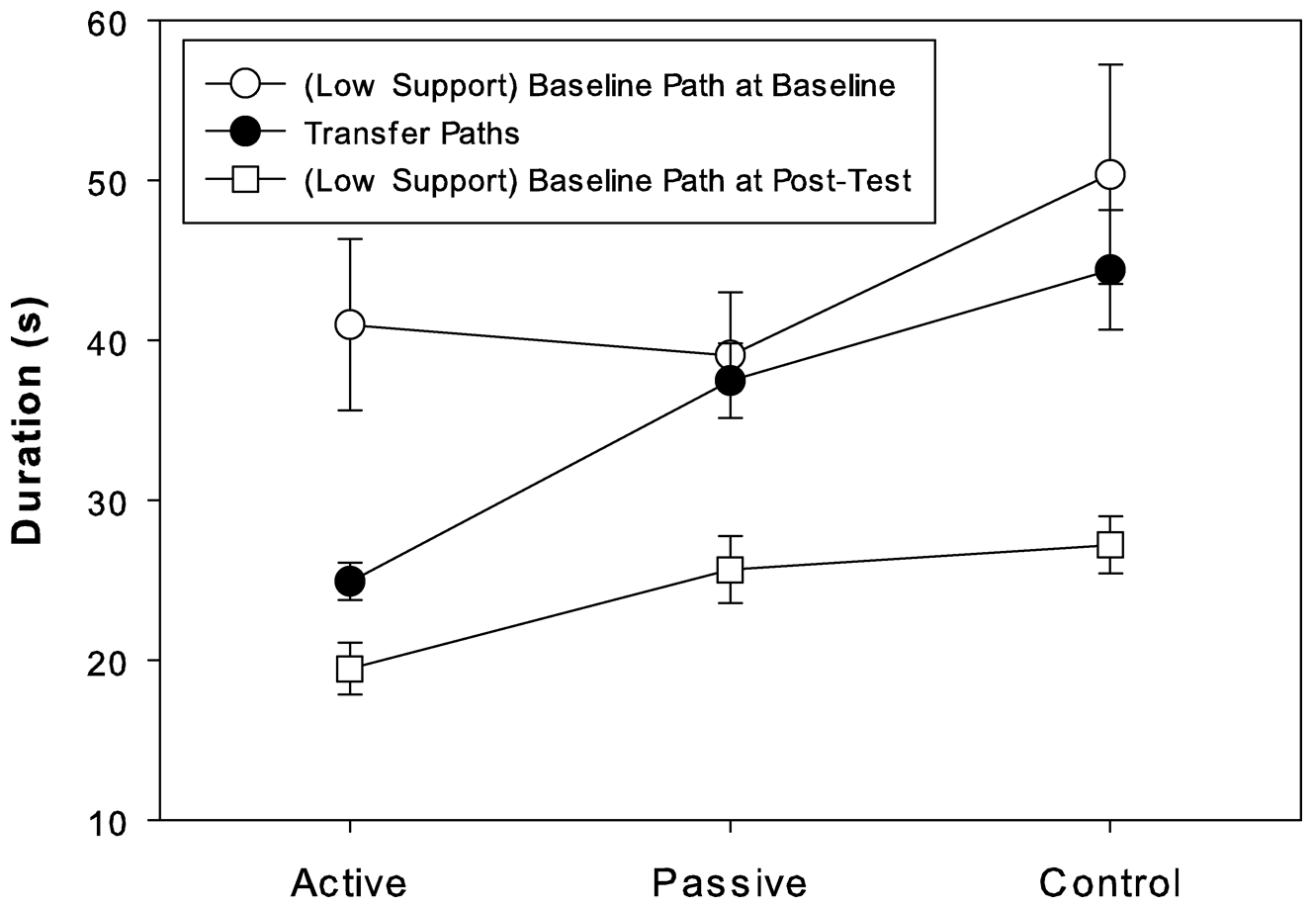
Mean trial durations (SE) for paths with low support levels by group and session/path type.

We then tested the generalization of training effects. As shown in Figure 4, any improvements from learning failed to transfer to the Transfer Paths for participants in the passive and control groups as compared to the active group, where Transfer trials exhibited significant improvement. As revealed by a single factor ANOVA on mean trial duration for the Transfer Paths, the active group performed the Transfer Paths faster than the other groups (F(2,33) = 13.9, p<0.001). Transfer trials were performed with the lowest level of support and thus, were to be compared to Baseline trials at that lowest support level. In the passive and control groups, mean durations for Transfer trials were found to be not different from those comparable Baseline trials, but in the active group, they were found to be different. This was all revealed by single factor repeated-measures ANOVAs comparing post-training Transfer trials with Baseline trials at the same (lowest  = 0.13N) support level separately for each group (active: F(1,23) = 10.8, p<0.01; passive: p>0.6; control: p>0.1). However, all three groups yielded a significant difference when post-training Transfer trials were compared to Post-Training trials at the lowest support level (F(1,23)>21.0, p<0.001).

### Spatial measures

We also assessed the spatial aspects of performance. We expected that the number of times a participant would come off the wire path would change as a function of learning. We failed to find a difference among groups in this regard when we compared times off path at baseline and post training. All participants exhibited reduction in times off path. However, when we tested transfer trials in this way we found a significant difference as a function of group. The active training group yielded fewer times off path than either passive training or control groups, which were not different from one another. The pattern of results was the same when we next analyzed path lengths. Post training trials exhibited shorter path lengths than baseline, but no differences among groups. However, path lengths for transfer trials were significantly shorter for the active training group than the passive training and control groups that were, in turn, not different from one another.

First, we examined the number of times the stylus came off the target path each trial. In general, participants tended to come off of the path more frequently before versus after training (an average of 1.0 before training versus 0.05 after for the highest support level to 13.4 before training versus 6.4 after for the lowest support level). A mixed design ANOVA on learning scores (baseline – post-training) revealed that the frequency off path varied as a function of support level (support level: F(5,165) = 18.0, p<0.001) but not group (group: p>0.4; group by support level interaction: p>0.2). However, when we analyzed Transfer trials, differences between groups appeared (F(2,69) = 6.1, p<0.005). Passive and control participants were much more likely to come off the path than the active group participants. The group means (and standard errors) were as follows: active  = 6.7 (0.6), passive  = 10.5 (1.1), and control  = 10.9 (1.0).

We then analyzed path lengths to confirm the findings for the measure of frequency off path. The pattern of results was the same as for frequency off path. In general, path lengths were longer before as compared to after training (138 cm before training versus 121 cm after for the highest support level (that is, spring stiffness  = 2.2N) to 251 cm before training versus 190 cm after for the lowest support level (spring stiffness  = 0.13N)). A mixed design ANOVA on the learning scores for path length revealed an effect of support level (F(5,165) = 8.0, p<0.001), but no differences as a function of group (group: p>0.6; group by support level interaction: p>0.5). Again however, when we analyzed Transfer trials, there were differences in path length between the groups (F(2,69) = 4.9, p<0.01). The group means (and standard errors) were as follows: active  = 196 cm (7.1 cm), passive  = 259 cm (19.0 cm), and control  = 238 cm (15.1 cm).

### Relations between temporal and spatial measures (showing how performance improved)

Next, we examined the relation between the temporal and spatial measures. The two spatial measures were related to one another, so we focused on the frequency off path measure as best reflecting the temporal challenge element of the task. We investigated whether change in frequency off path might have yielded faster performance of the task, that is, lower durations. Did the time to get back onto the path change or instead, did the speed of movement while remaining on the path change? To anticipate, we found that the latter was the case showing that improved performance was produced by improved prospective control, that is, participants better anticipated the path to be traced so as to be able to follow it successfully and faster without coming off it.

At baseline, the three groups were not different from one another, so we combined the data from the groups and regressed frequency off path against Duration. The linear relation was as follows: Duration  = 2.16× frequency off path +12.85 (r^2^ of 0.85). That is, each time a participant came off the path with the stylus, it added approximately 2 seconds to the 12.85 seconds it took participants to complete a trial on average at baseline.

Next, because the active group was different from the passive and control groups (which were not different from one another) after training, we performed the regression separately on the post-training data for (i) the active group and then (ii) the combined data of the passive and control groups. The linear relation for the active group was: Duration  = 2.16× frequency off path +7.6 (r^2^ of 0.82). The linear relation for the passive and control groups was as follows: Duration  = 1.92× frequency off path +11.22 (r^2^ of 0.68). We performed a multiple regression to test whether these respective slopes and intercepts were different [57]. (The two groups were coded as +/− 1 to test the intercepts and this vector was multiplied by the frequency off path vector to produce a third vector used to test the slopes.) The result was significant (r^2^ = 0.77, F(3, 426) = 485.2, p<.001) with reliably different Intercepts (t = 9.3, p<.001) and Slopes (t = 2.0, p<0.05). Despite the Slope difference of 0.24 s ( = 2.16 s – 1.92 s) per frequency off path, the group difference in durations shown in Figure 3 was reflected primarily in the Intercept difference of 3.62 seconds ( = 11.22 s – 7.60 s). Essentially, it took all participants about 2 seconds to get the stylus back onto the path every time they came off and this did not change greatly with training. Instead, active participants became faster at successfully moving the stylus along the path. This is evidence of improved prospective control. On average, participants in the active group were faster than the passive and control participants by 3.6 seconds per trial, and they improved as a result of active practice by 5.25 seconds per trial ( = 12.85 s – 7.60 s).

As shown in Table 1, this improved performance generalized to a reduction in the frequency off path achieved in the Transfer trials post-training. We compared the results for the Baseline Path at the lowest support level (0.13N) at both baseline and post-training with the results for the Transfer Paths. The Transfer mean was significantly different from the baseline mean (F(1, 11) = 7.7, p<0.02) for the active group, but not for the passive (p>0.2) or control (p>0.1) groups. This was despite the fact that post-training means were different from baseline (F(1, 11)>9.0, p<0.02 or better) for all three groups. Instead, for passive and control groups, the post-training and Transfer means were different (F(1, 11)>16.5, p<0.002). Thus, improvements in control achieved by participants in the active group allowed them to avoid coming off the paths often on both practiced and novel paths. The lack of active practice appears to have prevented the other groups from achieving this. Again, the improvement in performance reflected better prospective control. The active participants learned to anticipate the changes along the path so as to avoid coming off the path.

**Table 1:** Mean Frequency Off Path (SE) during Low Support by Group, Path, and Session.

Group	Baseline Path at Baseline (Level 6)	Baseline Path at Post-Test (Level 6)	Transfer Paths (Level 6)
Active	14.33 (2.90)	4.92 (0.61)	6.71 (0.85)
Passive	11.88 (1.25)	7.58 (1.08)	10.50 (1.38)
Control	14.12 (2.60)	6.92 (1.04)	10.94 (0.99)

### Additional control group

It was possible that the passive group were slower than the active group after training simply because they were trained to be slower i.e. the trial duration during training was slow. We therefore trained another group of 12 participants (5 females, 7 males, 18–35 years old, all right handed) in the passive (or haptic tracking) condition with faster trial durations but otherwise everything was identical to the main experiment. The initial passive group was trained with trials that were 15 s in duration. This second passive group was trained with trials of that were 6 s in duration (this duration was selected because it was faster than the average duration produced by the participants in the active group during training). We computed learning scores for this additional group and then compared performance with that of the original passive group to determine whether there were differences. There was no indication that the groups were different from each other. We performed a mixed design ANOVA testing the effect of group, support level, and the group by support level interaction for the Baseline path. We found:

1. trial duration: no effect of group (F(1,22) = 0.7, p>0.4), no group by support level interaction (F(5,110) = 1.0, p>0.4).
2. frequency off path: no effect of group (F(1,22) = 0.2, p>0.6), no group by support level interaction (F(5,110) = 0.5, p>0.7).

We performed single factor ANOVAs testing the effect of group for the Transfer paths. We found:

1. trial duration: no effect of group (F(1,46) = 1.8, p>0.2).
2. frequency off path: no effect of group (F(1,46) = 0.2, p>0.7).

Thus, we are confident that the differences between the active and passive groups were not explained by training tempo.

## Discussion

The goal of the current study was to investigate whether effective motor learning would be allowed by passive control, meaning in this case control lacking an active prospective perceptual component. We compared learning with practice in a passive (haptic tracking) task versus practice in an active prospective control task with variable support. The prediction was that only active prospective control would yield effective sensorimotor learning.

While the effectiveness of haptic tracking as a training method for sensori-motor learning has previously been tested [25], [51], it has not been tested in acquisition of a generalizable motor skill under conditions that allowed a rigorous comparison between active and passive training by prescribing movements precisely to ensure that the conditions are the same aside from the control regime used by the participants. The previous studies only tested the ability to produce a simple, strictly circular path of constant length and curvature with movement in a single, constant direction. The same simple movement was tested in baseline, training, and post training with no test of ‘transfer or generalization. The current study tested the ability to push a bead rapidly along an arbitrarily complex smooth 3D path consisting of variable length, curvature, and torsion. Although different (and more difficult) paths were tested to investigate generalization or transfer of the skill, on the one hand, the same paths were tested (in baseline, post training, and transfer phases) across the different training conditions, that is, passive (haptic tracking) and active (with variable support). Thus, potentially competing goals were achieved in the current study, that is, to study the acquisition of a generalizable motor skill under appropriately constrained and controlled conditions allowing strict comparison of different training methods.

The task employed in this study was ideally suited to testing this question because the task was representative of many manual tasks while nevertheless allowing direct comparison of active versus passive learning with movements that were the same in all other respects. The task was representative because it required the hand to be guided along a 3D path with compliance control, that is, control of the interactions between the hand and a constraint surface. The results showed an unambiguous advantage of active movement control over the passive condition, where the latter was passive in respect to a lack of prospective control (and not the quiescence of the musculature). We varied two parameters during training for the active group: level of attraction (stiffness) and path configuration. As such, we cannot attribute learning to just one factor beyond the fact that the training required active control. Optimal training could be produced by either one or the combination of the two. However, our findings indicate that passively experiencing a desired movement pattern is not an effective route to learning – the individual needs to perform prospective control of the trajectories during practice. This finding is predicted both by action theory (e.g. [14], [15]) and by computational models of motor control (e.g. [16]).

We did not predict successful learning in the passive groups, but learning did seem to occur. Nevertheless, the amount of learning was less than observed in the active group. Furthermore, the learning observed on the practice paths failed to transfer effectively to novel paths (in contrast to the active group). The key to understanding these results was a comparison between the passive groups and the control group. The participants in the control group only performed trials in the baseline and post-training condition and were not exposed to any training trials. At baseline, participants performed two trials at each of the six support levels for a total of 12 trials. Of central importance is the fact that those trials necessarily had to be performed actively. Thus, although the participants in the control group did not perform any specific training trials, they performed 12 active trials at baseline and this was also the case for the passive groups. The improvement exhibited by the passive groups was identical to that shown by the controls. The conclusion is, therefore, that it was the active practice in the baseline trials that yielded the motor learning observed in the passive and control groups. Moreover, extensive passive training yielded no measurable improvements relative to the control group performance (i.e. there were no differences between the passive and control groups on any measure). Thus, the form of passive training deployed in this study can be concluded to be ineffective in producing motor learning.

Our analyses of both the temporal and spatial characteristics of performance revealed the specific nature of the improvement in performance exhibited by the participants who practiced with fully active movements. Those participants improved in their continuous control of the stylus moving along the required path so as to reduce the frequency with which they mistakenly left the target path. We found that the decreases in times for completing a trial were not yielded by quicker return to the target path after the stylus had left the path. Instead, the participants exhibited improved prospective control that better anticipated the path (its 3D curvature and torsion) so as to successfully maintain contact with and follow that path and avoid costly departures from the target path. Practice that allowed and supported active prospective control yielded progressive improvements in prospective control and thus, effective sensori-motor learning.

Our findings have major implications for the design of robotic systems being built to help improve upper limb function in conditions such as stroke, cerebral palsy and developmental coordination disorder. One of the goals of robotic research is to develop systems that can generate substantial forces around the shoulder and elbow joints (or at an endpoint attached to the patient’s hand) to help stroke survivors [58]. The results from our study suggest that the provision of sufficient force to move the arm passively might not be necessary if the goal of the therapy is to improve movement control (though passive movements might be beneficial in treating spasticity as a precursor to treating control deficits). The problem faced by designers of therapeutic robotic systems for individuals with severe disability is that assistive forces are required to help patients achieve movement goals, but, as shown by the current results combined with others reviewed in the introduction, if the assistance is too prescriptive then the training becomes passive in nature, and the therapeutic value is lost. Instead, the current results combined with those of Marchal-Crespo et al. [52] suggest that forms of variable or graduated ‘corrective assistance’ (assistance applied only when errors are made) experienced by active learners is a good approach when designing effective robot therapy systems. Providing more support at the outset and then gradually reducing the level of support as performance improves yields both good sensori-motor learning and good self-efficacy [53]. The learner constantly succeeds, and thus remains motivated to persist in training.

While our approach to ‘corrective assistance’ was somewhat similar to that used by Marchal-Crespo et al. [52], there were important differences. To force steering back to a desired target trajectory, Marchal-Crespo et al. used an applied force that was proportional to errors once they exceeded a criterion value. In the current study, a spring-force (of adjustable stiffness) held a user-controlled stylus onto a virtual 3D wire path. This force was experienced as a magnetic attraction that allowed the user to focus on producing movement strictly along the targeted 3D path (that is, the wire). This, in turn, entailed the development of (1) kinesthetic sensitivity to the wire as a constraint surface (and thus, good compliance control), and (2) visual sensitivity to the 3D shape of the wire (and thus, good prospective control). The best strategy for performing the resulting task was to move in a compliant manner while looking ahead to anticipate curves in the wire to allow the wire to guide the movement. The approach used in Marchal-Crespo et al. [52] also seemed to yield better prospective control in that task, but not better compliance control. Our approached yielded both simultaneously as uniquely relevant to our task.

A major motivation for conducting this study was to determine whether a passive training regime might be an effective approach for children with Developmental Coordination Disorder (DCD) as it has the advantage of requiring less effort from the children and allows the child to experience a successful movement trajectory. The results show that our initial decision to use an active training approach was sensible. Our previous work with children showed that training that supports, but requires active prospective control, improves performance in children with motor problems [53]. We designed the task in part to capture aspects of handwriting so that we might train children with DCD to improve their handwriting skill. The active control task taps into many core motor requirements of handwriting skill while avoiding the non-motor components that might be confounding factors (e.g. language skills). The ability to generate the appropriate levels of force, produce a smooth trajectory and use visual anticipatory information to avoid making errors are all essential elements within handwriting but also underpin many other skills. Common tasks with these characteristics include drawing, using cutlery, tying shoelaces, painting and any task requiring the manipulation of a surface with a handheld stylus. While the task we developed was representative in many useful ways, it was also novel so it required the acquisition of skill if participants were to become proficient at the task.

In conclusion, our results suggest that active generation and control of limb movements is required for effective motor learning. Passive forms of training did not yield good learning. What ‘active’ means is not simply stimulated musculature and active sensorimotor loops. These aspects were present in our passive training task, but these factors alone failed to yield good learning. Active means prospective control of limb movement trajectories, control in which perceptual information is used to anticipate the required trajectory and to overcome potential inaccuracy and instability caused by biologically determined delays in control. Our results present a challenge when developing robotic interventions for people with movement disorders. Nevertheless, the results obtained from the active group in this study (combined with those of Marchal-Crespo et al. [52]) suggest that corrective assistance (only providing assistance when the hand leaves a pre-defined zone) is an effective technique that has great potential to meet the goals of providing assistance whilst allowing active control.
